# The costs of overweight and obesity-related diseases in the Brazilian public health system: cross-sectional study

**DOI:** 10.1186/1471-2458-12-440

**Published:** 2012-06-18

**Authors:** Luciana Bahia, Evandro Silva Freire Coutinho, Laura Augusta Barufaldi, Gabriela de Azevedo Abreu, Thainá Alves Malhão, Camila Pepe Ribeiro de Souza, Denizar Vianna Araujo

**Affiliations:** 1 Internal Medicine Department, State University of Rio de Janeiro (UERJ) and National Institute of Science and Technology for Health Technology Assessment (IATS) – CNPq/Brazil, Blv. 28 de Setembro 77 – 3rd floor – room 329, Vila Isabel, ZIP 20551-030, Rio de Janeiro/RJ, Brazil; 2 National School of Public Health, Oswaldo Cruz Foundation (Fiocruz), Leopoldo Bulhões Street, 1480, Manguinhos, ZIP 21041-210, Rio de Janeiro/RJ, Brazil; 3 Institute for Studies in Public Health, Federal University of Rio de Janeiro (UFRJ), Blv. Horácio Macedo, No number - Fundão Island, University City, ZIP 21941-598, Rio de Janeiro/RJ, Brazil; 4 City Department of Health and Civil Defense of Rio de Janeiro (SMSDC-RJ), Afonso Cavalcanti Street, 455, 8th floor, room 801, New Town, ZIP 20211-110, Rio de Janeiro/RJ, Brazil; 5 Medinsight Decisions in Health Care, Hollywood Street, 330, Brooklin, ZIP 04564-040, São Paulo/SP, Brazil

**Keywords:** Overweight, Obesity, Costs, Brazil

## Abstract

**Background:**

Obesity is a major global epidemic and a burden to society and health systems. It is well known risk factor for a number of chronic medical conditions with high morbidity and mortality. This study aimed to provide an estimate of the direct costs associated to outpatient and inpatient care of overweight and obesity related diseases in the perspective of the Brazilian Health System (SUS).

**Methods:**

Population attributable risk (PAR) was calculated for selected diseases related to overweight and obesity and with the following parameters: Relative risk (RR) ≥ 1.20 or RR ≥1.10 and < 1.20, but important problem of public health due its high prevalence. After a broad search in the literature, two meta-analysis were selected to provide RR for PAR calculation. The prevalence rates of overweight and obesity in Brazilians with ≥18 years were obtained from large national survey. The national health database (DATASUS) was used to estimate the annual cost of the Brazilian Unified Health System (SUS) with the diseases included in the analysis. The extracted values were stratified by sex, type of service (inpatient or outpatient care) and year. Data were collected from 2008 to 2010 and the results reflect the average of 3 years. Brazilian costs were converted into US dollars during the analysis using a purchasing power parity basis (2010).

**Results:**

The estimated total costs in one year with all diseases related to overweight and obesity are US$ 2,1 billion; US$ 1,4 billion (68.4% of total costs) due to hospitalizations and US$ 679 million due to ambulatory procedures. Approximately 10% of these cost is attributable to overweight and obesity.

**Conclusion:**

The results confirm that overweight and obesity carry a great economic burden for Brazilian health system and for the society. The knowledge of these costs will be useful for future economic analysis of preventive and treatment interventions.

## Background

Over the past several decades, obesity has grown into a major global epidemic. World Health Organization (WHO) estimated that globally in 2005, approximately 1.6 billion adults were overweight and at least 400 million adults were obese. They also predicted that by 2015, approximately 2.3 billion adults would be overweight and more than 700 million would be obese [1]. In Brazil, two national surveys of adult population showed that overweight and obesity prevalence rates has increased over the past 4 years, from 43% to 48.1% and from 11% to 15% for overweight and obesity respectively [2,3].

Overweight and obesity are known risk factors for a number of chronic medical conditions, like cancer, diabetes, or heart disease, that in turn are primary drivers of health care spending, disability, and death. The estimated numbers of obesity-attributed cancers are large and include common cancer as pancreas, colorectum, breast, and endometrium. In the United States the impact of obesity on the related incident cancers in 2007 account for about 6% of all cancers in that country [4]. A study reported that obesity-related morbidity is greater than that associated with smoking, drinking, or poverty in the United States [5].

The economic costs of obesity have raised considerable attention in recent years. The cost, or burden, of an illness can be measured by the financial impact of related diseases on the health system (direct costs) and by the loss of productivity and quality of life (indirect costs) to society and individuals. Obesity presents a major health challenge, especially in developing countries as Brazil and the costs are substantial, although unknown in most health systems.

The primary objective of this study is to provide an estimate of the direct costs associated to outpatient and inpatient care of overweight and obesity-related diseases in the perspective of the Brazilian Health System (SUS).

## Methods

### Population attributable risk

Population attributable risk (PAR) is the proportion of the incidence of a disease in the population that is due to exposure to a particular risk factor. It is the incidence of a disease in the population that would be eliminated if exposure were suppressed. This section describes the procedures for the selection of diseases and to estimate the PAR used for the cost study.

Two groups of exposure were of interest for estimating the PAR: individuals with overweight (body mass index-BMI 25-29.9 Kg/m^2^) and obesity (BMI ≥ 30 Kg/m^2^).

The related diseases were selected for calculating PAR if their associations with overweight and obesity have the following parameters: Relative risk (RR) ≥ 1.20 or Relative risk (RR) ≥1.10 and < 1.20, but important problem of public health due its high prevalence.

The estimate of PAR was done by Levin’s formula, defined as the proportion of all cases that would not have occurred if the exposure had been absent [6].

(1) $$PAR=\frac{Pe\left(RR-1\right)}{Pe\left(RR-1\right)+1}$$

where *P*_*e*_ is the prevalence of exposure and *RR* the relative risk.

### Sources of relative risks estimates

First, we searched for meta-analyses presenting RR estimates associated to the presence of overweight and obesity. After identifying the most recent meta-analyses, we looked for large individual studies published after the search period covered by the meta-analyses. We conducted a literature search in two databases, Medline and Scopus. The search always contained two blocks of concepts: one of the descriptors of exposure (overweight or obesity) and the other descriptors related to the selected outcomes. The Table 1 presents the relative risks and respective 95% confidence intervals, by gender, for the selected diseases (for overweight and obesity).

**Table 1:** Relative risks and respective 95% confidence intervals, by gender, for selected diseases

Disease	Overweight	Obesity
Colon-rectum cancer	M: 1.48 (1.23-1.79)	M: 1.95 (1.51-2.51)
	W: 1.55 (1.30-1.86)	W: 1.49 (1.21-1.82)
Ovary cancer	W: 1.29 (1.12-1.23)	W :1.47 (1.13-1.91)
Endometrium cancer	W: 1.90 (1.53-2.36)	W: 3.39 (2.51-4.58)
Diabetes II	M: 2.29 (1.98-2.64)	M: 5.36 (4.32-6.65)
	W: 3.64 (2.93-4.52)	W: 10.47 (7.31-15.0)
Arterial hypertension	M: 2.34 (1.85-2.98)	M :5.93 (4.39-8.0)
	W: 2.04 (1.33-3.12)	W: 3.48 (2.12-5.71)
Stroke	M: 1.23 (1.13-1.34)	M: 1.51 (1.33-1.72)
	W: 1.15 (1.00-1.32)	W: 1.49 (1.27-1.74)
Ischemic Heart Disease	M: 1.29 (1.18-1.41)	M: 1.72 (1.51-1.96)
	W: 1.14 (0.88-1.48)	W: 1.91 (1.45-2.50)
Congestive Heart Failure (men)	M: 1.36 (1.01-1.83)	M: 1.80 (1.27-2.56)
	W: not included	W: 1.78 (1.07-2.95)
Asthma	M: 1.20 (1.08-1.33)	M: 1.43 (1.14-1.79)
	W: 1.25 (1.05-1.49)	W: 1.78 (1.36-2.32)
Osteoarthritis (knee and hip)	M: 2.01 (1.92-2.09)	M: 2.47 (2.27-2.70)
	W: 1.80 (1.75-1.85)	W: 1.96 (1.88-2.04)
Pancreas cancer	M: not included	M: 2.74 (1.60-4.67)
	W: not included	W: 1.57 (1.06-2.33)
Kidney cancer	M: not included	M: 1.97 (1.20-3.22)
	W: not included	W: 1.99 (1.42-2.78)
Gallbladder cancer	M: 1.15 (1.01-1.30)	M: 1.35 (1.09-1.68)
	W: 1.15 (1.01-1.30)	W: 1.88 (1.66-2.13)
Post-menopausal breast cancer	W: 1.11 (1.01-1.22)	W: 1.17 (1.04-1.32)

M:men; W:women.

The quality evaluation of the meta-analyses was done by AMSTAR inventory, that scored the methodological aspects of the meta-analyses used in this study [7]. The meta-analyses done by Guh et al. [8] and Larson et al. [9] were selected to provide RR for PAR calculation.

### Sources of population estimates for the exposures

The prevalence rates of overweight and obesity in individuals with ≥18 years were obtained from a recent and large national survey called VIGITEL study [3], which assessed by telephone interviews 54.339 individuals, 20.764 men and 33.575 women. This study made use of self-reported data on weight and height to calculate the body mass index (BMI). The survey presents the prevalence for overweight and obesity separately, stratified by gender and state.

### Cost estimates

DATASUS database was used to estimate the annual cost of the Brazilian Unified Health System (SUS) with diseases that were included in the analysis [10]. This database provides the reimbursed values by the government for public health care organizations that provide health care (inpatient and outpatient) needed for treatment and monitoring of these diseases. The values extracted from DATASUS were stratified by sex, type of service (inpatient or outpatient care) and year. Data were collected from 2008 to 2010 and the results reflect the average of 3 years. As PAR was calculated stratified by Brazil's capitals, the values were extracted from DATASUS initially by capital and later by state.

Brazilian costs were converted into US dollars during the analysis using a purchasing power parity basis (PPP 2010: US$ 1 = R$ 1.7) [11].

## Results

The influence of overweight and obesity on selected diseases varies a lot, from 2% to breast cancer (overweight) to 66.43% to type 2 diabetes mellitus (obesity). The calculated overweight and obesity PAR are listed in Tables 2 and 3.

**Table 2:** Overweight PAR by gender and Brazilian capitals, in percentage, for the selected diseases

Brazilian Capitals	Colorectal cancer		EC	PBC	Ovarian cancer	Gallbladder cancer		Asthma		IHD		Osteoarthritis		Diabetes		Hypertension		Stroke		CHF
	M	W	M	W	W	M	W	M	W	M	W	M	W	M	W	M	W	M	W	M
Aracajú	14.14	14.53	21.76	3.29	8.22	4.89	4.43	6.42	7.17	9.05	4.15	25.73	19.82	30.67	44.93	31.49	24.32	7.31	4.43	10.99
Belém	14.87	12.89	19.49	2.87	7.24	5.18	3.88	6.79	6.30	9.55	3.63	26.88	17.71	31.95	41.53	32.78	21.86	7.73	3.88	11.59
Belo Horizonte	14.31	13.63	20.53	3.06	7.68	4.96	4.13	6.51	6.69	9.17	3.86	26.01	18.67	30.98	43.11	31.80	22.99	7.41	4.13	11.13
Boa Vista	13.89	12.68	19.20	2.82	7.11	4.80	3.81	6.30	6.19	8.88	3.56	25.34	17.44	30.24	41.07	31.05	21.54	7.17	3.81	10.79
Campo Grande	15.97	14.00	21.04	3.15	7.91	5.61	4.25	7.34	6.89	10.30	3.98	28.57	19.15	33.81	43.87	34.67	23.54	8.35	4.25	12.48
Cuiabá	14.42	13.43	20.24	3.01	7.56	5.00	4.06	6.56	6.59	9.24	3.80	26.17	18.41	31.17	42.68	31.99	22.68	7.47	4.06	11.22
Curitiba	15.43	12.09	18.37	2.68	6.76	5.39	3.61	7.06	5.88	9.93	3.38	27.74	16.67	32.89	39.76	33.74	20.63	8.04	3.61	12.03
Florianópolis	15.67	12.30	18.67	2.73	6.89	5.49	3.68	7.18	5.99	10.09	3.45	28.10	16.94	33.30	40.23	34.15	20.96	8.17	3.68	12.23
Fortaleza	13.42	16.41	24.32	3.78	9.38	4.62	5.08	6.07	8.19	8.56	4.76	24.60	22.22	29.41	48.52	30.21	27.08	6.92	5.08	10.42
Goiânia	16.17	12.26	18.61	2.72	6.86	5.69	3.67	7.44	5.97	10.44	3.43	28.88	16.89	34.15	40.14	35.01	20.90	8.46	3.67	12.64
João Pessoa	15.80	13.18	19.90	2.95	7.41	5.54	3.98	7.25	6.45	10.18	3.72	28.31	18.09	33.53	42.15	34.38	22.30	8.25	3.98	12.34
Macapá	15.87	12.34	18.73	2.74	6.91	5.57	3.70	7.29	6.02	10.23	3.46	28.41	17.00	33.64	40.33	34.50	21.03	8.29	3.70	12.39
Maceió	15.22	13.22	19.96	2.96	7.44	5.31	3.99	6.96	6.48	9.78	3.73	27.42	18.14	32.54	42.24	33.38	22.37	7.92	3.99	11.87
Manaus	14.91	12.93	19.55	2.88	7.26	5.19	3.89	6.80	6.32	9.57	3.64	26.94	17.76	32.01	41.62	32.85	21.92	7.74	3.89	11.61
Natal	14.14	14.28	21.43	3.23	8.08	4.89	4.35	6.42	7.04	9.05	4.07	25.73	19.51	30.67	44.44	31.49	23.96	7.31	4.35	10.99
Palmas	11.88	9.91	15.25	2.15	5.48	4.04	2.91	5.32	4.76	7.53	2.72	22.11	13.79	26.60	34.55	27.35	17.22	6.07	2.91	9.19
Porto Alegre	15.80	15.09	22.52	3.43	8.56	5.54	4.62	7.25	7.47	10.18	4.33	28.31	20.53	33.53	46.03	34.38	25.15	8.25	4.62	12.34
Porto Velho	16.31	13.05	19.72	2.92	7.34	5.74	3.93	7.51	6.39	10.53	3.68	29.08	17.93	34.37	41.88	35.23	22.11	8.54	3.93	12.75
Recife	14.31	14.20	21.32	3.20	8.03	4.96	4.32	6.51	7.00	9.17	4.04	26.01	19.41	30.98	44.28	31.80	23.84	7.41	4.32	11.13
Rio Branco	18.31	13.39	20.19	3.00	7.53	6.55	4.04	8.54	6.56	11.93	3.79	32.05	18.35	37.59	42.59	38.49	22.61	9.70	4.04	14.39
Rio de Janeiro	16.88	14.45	21.65	3.27	8.18	5.97	4.40	7.80	7.13	10.93	4.12	29.93	19.72	35.30	44.77	36.18	24.20	8.87	4.40	13.22
Salvador	13.35	12.72	19.26	2.83	7.14	4.59	3.82	6.03	6.21	8.52	3.58	24.48	17.49	29.28	41.16	30.08	21.61	6.88	3.82	10.36
São Luis	13.78	12.13	18.43	2.69	6.79	4.76	3.63	6.24	5.90	8.81	3.39	25.17	16.72	30.05	39.85	30.85	20.70	7.11	3.63	10.70
São Paulo	15.01	14.32	21.48	3.24	8.10	5.23	4.36	6.86	7.06	9.64	4.08	27.10	19.56	32.19	44.52	33.03	24.02	7.80	4.36	11.70
Teresina	15.77	10.22	15.70	2.23	5.66	5.53	3.01	7.24	4.92	10.16	2.82	28.26	14.21	33.47	35.34	34.32	17.71	8.23	3.01	12.31
Vitória	15.43	11.32	17.27	2.49	6.30	5.39	3.36	7.06	5.48	9.93	3.15	27.74	15.65	32.89	37.98	33.74	19.44	8.04	3.36	12.03
Distrito Federal	19.04	10.57	16.21	2.31	5.87	6.85	3.12	8.93	5.10	12.44	2.92	33.11	14.68	38.73	36.21	39.64	18.27	10.13	3.12	14.99

Population Attributable Risk: PAR; M: Men; W: Women; EC: Endometrial Cancer; PBC: Postmenopausal Breast cancer; CHF: Congestive Heart Failure; IHD: Ischemic Heart Disease.

**Table 3:** Obesity PAR by gender and Brazilian capitals, in percentage, for the selected diseases

Brazilian capitals	Colorectal cancer		EC	PBC	Ovarian cancer	Pancreatic cancer		Kidney Cancer		Gallbladder cancer		Asthma		IHD		CHF		Osteoarthritis		Diabetes		Hypertension		Stroke	
	M	W	W	W	W	M	W	M	W	M	W	M	W	M	W	M	W	M	W	M	W	M	W	M	W
Aracajú	12.18	6.72	26.00	2.44	6.46	20.26	7.73	12.41	12.70	4.86	11.45	5.91	10.29	9.51	11.80	10.46	10.29	17.67	12.37	38.90	58.20	41.85	26.72	6.93	6.72
Belém	13.05	6.16	24.26	2.23	5.92	21.56	7.10	13.29	11.71	5.24	10.55	6.36	9.46	10.21	10.87	11.22	9.46	18.85	11.40	40.79	55.93	43.79	24.94	7.46	6.16
Belo Horizonte	10.39	6.33	24.80	2.29	6.09	17.51	7.29	10.58	12.02	4.10	10.83	4.98	9.72	8.07	11.16	8.89	9.72	15.21	11.70	34.72	56.65	37.56	25.50	5.86	6.33
Boa Vista	12.18	7.65	28.77	2.79	7.36	20.26	8.79	12.41	14.33	4.86	12.95	5.91	11.65	9.51	13.33	10.46	11.65	17.67	13.96	38.90	61.54	41.85	29.53	6.93	7.65
Campo Grande	12.18	8.35	30.77	3.07	8.04	20.26	9.59	12.41	15.55	4.86	14.07	5.91	12.67	9.51	14.48	10.46	12.67	17.67	15.15	38.90	63.79	41.85	31.57	6.93	8.35
Cuiabá	15.29	8.27	30.54	3.03	7.96	24.85	9.49	15.56	15.41	6.24	13.94	7.55	12.55	12.03	14.34	13.19	12.55	21.83	15.01	45.31	63.54	48.37	31.33	8.83	8.27
Curitiba	14.60	7.81	29.25	2.86	7.52	23.85	8.98	14.86	14.62	5.93	13.21	7.18	11.89	11.47	13.60	12.59	11.89	20.92	14.24	43.97	62.10	47.02	30.02	8.41	7.81
Florianópolis	12.84	6.16	24.26	2.23	5.92	21.24	7.10	13.07	11.71	5.15	10.55	6.25	9.46	10.04	10.87	11.03	9.46	18.56	11.40	40.33	55.93	43.32	24.94	7.33	6.16
Fortaleza	17.09	6.89	26.52	2.50	6.63	27.41	7.92	17.39	13.00	7.06	11.73	8.53	10.54	13.51	12.08	14.79	10.54	24.18	12.66	48.62	58.85	51.69	27.25	9.96	6.89
Goiânia	10.00	5.60	22.43	2.02	5.38	16.91	6.45	10.19	10.70	3.93	9.62	4.79	8.62	7.77	9.92	8.56	8.62	14.68	10.41	33.78	53.40	36.58	23.08	5.63	5.60
João Pessoa	12.18	8.15	30.20	2.99	7.84	20.26	9.35	12.41	15.20	4.86	13.74	5.91	12.37	9.51	14.14	10.46	12.37	17.67	14.80	38.90	63.15	41.85	30.98	6.93	8.15
Macapá	11.89	8.06	29.96	2.95	7.76	19.81	9.26	12.11	15.05	4.73	13.61	5.75	12.25	9.28	14.01	10.20	12.25	17.27	14.66	38.24	62.90	41.18	30.74	6.75	8.06
Maceió	11.59	5.95	23.57	2.15	5.72	19.36	6.85	11.81	11.32	4.61	10.19	5.60	9.14	9.04	10.51	9.94	9.14	16.86	11.02	37.57	54.99	40.49	24.24	6.58	5.95
Manaus	12.98	8.88	32.23	3.27	8.55	21.46	10.19	13.22	16.46	5.21	14.90	6.32	13.44	10.16	15.33	11.16	13.44	18.75	16.04	40.64	65.33	43.63	33.04	7.41	8.88
Natal	13.27	7.77	29.13	2.84	7.48	21.88	8.93	13.51	14.55	5.33	13.15	6.47	11.83	10.39	13.53	11.41	11.83	19.14	14.17	41.24	61.96	44.25	29.90	7.59	7.77
Palmas	11.81	4.67	19.29	1.67	4.49	19.70	5.39	12.03	9.01	4.70	8.09	5.72	7.24	9.22	8.34	10.14	7.24	17.17	8.76	38.07	48.64	41.01	19.87	6.71	4.67
Porto Alegre	12.18	7.31	27.79	2.66	7.03	20.26	8.41	12.41	13.75	4.86	12.41	5.91	11.16	9.51	12.78	10.46	11.16	17.67	13.39	38.90	60.39	41.85	28.53	6.93	7.31
Porto Velho	11.96	9.29	33.31	3.43	8.94	19.92	10.64	12.18	17.14	4.77	15.53	5.79	14.02	9.33	15.98	10.27	14.02	17.37	16.71	38.40	66.43	41.35	34.14	6.80	9.29
Recife	16.10	6.85	26.39	2.49	6.59	26.01	7.88	16.38	12.93	6.60	11.66	7.99	10.47	12.70	12.01	13.91	10.47	22.90	12.59	46.83	58.69	49.90	27.11	9.34	6.85
Rio Branco	11.59	9.45	33.73	3.49	9.10	19.36	10.83	11.81	17.41	4.61	15.79	5.60	14.25	9.04	16.24	9.94	14.25	16.86	16.98	37.57	66.86	40.49	34.57	6.58	9.45
Rio de Janeiro	11.89	8.27	30.54	3.03	7.96	19.81	9.49	12.11	15.41	4.73	13.94	5.75	12.55	9.28	14.34	10.20	12.55	17.27	15.01	38.24	63.54	41.18	31.33	6.75	8.27
Salvador	7.06	6.38	24.94	2.31	6.13	12.22	7.34	7.20	12.10	2.72	10.90	3.33	9.78	5.45	11.23	6.02	9.78	10.52	11.77	25.86	56.83	28.28	25.64	3.92	6.38
São Luis	10.77	5.03	20.52	1.80	4.83	18.10	5.80	10.97	9.66	4.26	8.68	5.18	7.77	8.38	8.95	9.22	7.77	15.73	9.39	35.64	50.56	38.50	21.13	6.08	5.03
São Paulo	11.96	7.10	27.16	2.58	6.83	19.92	8.17	12.18	13.38	4.77	12.07	5.79	10.85	9.33	12.43	10.27	10.85	17.37	13.03	38.40	59.63	41.35	27.90	6.80	7.10
Teresina	12.40	5.86	23.29	2.11	5.63	20.59	6.75	12.63	11.17	4.96	10.05	6.02	9.01	9.69	10.36	10.65	9.01	17.97	10.87	39.38	54.60	42.35	23.95	7.06	5.86
Vitória	12.25	7.31	27.79	2.66	7.03	20.37	8.41	12.48	13.75	4.89	12.41	5.95	11.16	9.57	12.78	10.52	11.16	17.77	13.39	39.06	60.39	42.02	28.53	6.97	7.31
Distrito Federal	8.20	4.54	18.82	1.62	4.36	14.06	5.24	8.36	8.76	3.19	7.86	3.88	7.03	6.34	8.11	6.99	7.03	12.14	8.52	29.07	47.88	31.67	19.39	4.57	4.54

Population Attributable Risk: PAR; M: Men; W: Women; EC: Endometrial Cancer; PBC: Postmenopausal Breast cancer; IHD: Ischemic Heart Disease; CHF: Congestive Heart Failure.

The estimated total costs in one year with all diseases related to overweight and obesity are US$ 2,152,102,171. Hospitalizations account to US$ 1,472,742,952, 68.4% of total costs and ambulatory procedures account to US$ 679,353,348 (Table 4).

**Table 4:** Hospitalization, ambulatory, and total costs with estimated attributable costs of overweight and obesity related-diseases

	Hospitalization costs*	Hospitalization costs attributable to risk factors(%)	Ambulatory costs* (medical visits, exams, procedures)	Ambulatory costs attributable to risk factors(%)	Total costs* Outpatient and Inpatient care	Total costs attributable to risk factors (%)
	**WOMEN**		**WOMEN**		**WOMEN**	
Obesity	US$ 341,5 million	US$ 46,3 million (13.58%)	US$ 253,5 million	US$ 17,6 million (6.96%)	US$ 595 million	US$ 64 million (10.76%)
Overweight	US$ 262,8 million	US$ 20,2 million (7.69%)	US$ 247,2 million	US$ 15,4 million (6.28%)	US$ 510,1 million	UD$ 35,7 million (7.00%)
Obesity + overweight	US$ 604,3 million	US$ 66,6 million (11.02%)	US$ 500,8 million	US$ 33,1 million (6.62%)	US$ 1,1 billion	US$ 99,7 million (9.02%)
	**MEN**		**MEN**		**MEN**	
Obesity	US$ 435,6 million	US$ 47,2 million (10.86%)	US$ 91,9 million	US$ 12,4 million (13.53%)	US$ 527,6 million	US$ 59,7 million (11.32%)
Overweight	US$ 432,6 million	US$ 49,2 million (11.38%)	US$ 86,5 million	US$ 12,3 million (14.30%)	US$ 519,2 million	US$ 61,6 million (11.87%)
Obesity + overweight	US$ 868,3 million	US$ 96,5 million (11.12%)	US$ 178,5 million	US$ 24,8 million (13.90%)	US$ 1 billion	US$ 121,3 million (11.59%)
	**WOMEN AND MEN**		**WOMEN AND MEN**		**WOMEN AND MEN**	
Obesity	US$ 777,2 million	US$ 93,6 million (12.05%)	US$ 345,5 million	US$ 30 million (8.71%)	US$ 1,1 billion	US$ 123,7 million (11.02%)
Overweight	US$ 695,5 million	US$ 69,4 million (9.99%)	US$ 333,8 million	US$ 27,8 million (8.34%)	US$ 1 billion	US$ 97,3 million (9.45%)
Obesity + overweight	US$ 1,4 billion	US$ 163,1 million (11.08%)	US$ 679,3 million	US$ 57,9 million (8.53%)	US$ 2,1 billion	US$ 221 million (10.27%)

* Three years average (2008 to 2010) of costs related to selected diseases (Table 1).

Applying the PAR, overweight and obesity contribute with percentages between 6.28% to 14.3% of these costs according to sex, inpatient or outpatient care and presence of overweight or obesity.

Obesity attributable hospitalizations costs were higher among men (US$ 47 million *vs.* US$ 46 million), although PAR were lower than in women. The inverse situation was seen regarding outpatient costs, with much higher PAR but lower costs among men (US$ 12 million *vs.* US$ 18 million).

The analysis stratified by groups of diseases showed that the largest costs (out and inpatient care in both sexes) were due to cardiovascular disease (US$ 747 million) followed by overweight and obesity related neoplasms (US$ 299.8 million, mainly in women 79.5%), asthma (US$ 34 million), diabetes mellitus (US$ 23.7 million) and osteoarthritis (US$ 3.9 million) (Table 5). The major part of costs with cardiovascular diseases was due to coronary artery disease (60.5%).

**Table 5:** Annual costs separated by group of diseases (women and men)

Group of diseases		Ambulatory costs	Hospitalization costs	Total costs
Cardiovascular	All cardiovascular	US$ 87,4 million	US$ 664,0 million	US$ 751,4 million
	Coronary artery disease	US$ 65,1 million	US$ 389,7 million	US$ 454,9 million
	Cardiac failure	US$ 1,5 million	US$ 158,4 million	US$ 159,9 million
	Arterial hypertension	US$ 13,5 million	US$ 21,9 million	US$ 35,5 million
	Stroke	US$ 7,1 millions	US$ 93,9 million	US$ 101,0 million
Neoplasms*		US$ 239,6 million	US$ 60,1 million	US$ 299,8 million
Asthma		US$ 12,4 million	US$ 21,6 million	US$ 34,1 million
Diabetes Mellitus**		US$ 851,715 thousand	US$ 21,8 million	US$ 23,7 million
Osteoarthritis***		US$ 3,9 million	US$ 5,8 million	US$ 9,7 million

*Overweight and obesity related neoplasms **only as first diagnosis ***only knee and hip.

## Discussion

The estimated costs of diseases related to overweight and obesity reach almost US$ 2.1 billion in one year. Using population attributable risk factor we could estimate that approximately 10% of these cost is attributable to overweight and obesity. The estimates of direct costs reviewed here may generally be conservative, probably underestimated. The reimbursement rates provided by the Brazilian healthcare system (SUS) are widely recognized as poor estimates of the true costs of health care as shown by some cost-of-illness studies in Brazil, showing much higher costs than the reimbursed values [12,13]. If indirect costs such as days lost to sickness, premature mortality, out-of-pocket and home care expenses had been included, the figure would have been far higher. Furthermore, this study focused only on the cost of care provided at public hospitals, the overall cost in Brazil will clearly be higher than reported in this study if also private health care spending had been included.

Costs for women were greater than for men, mainly because of higher ambulatory expenditures (73.3% *vs* 26.7% of total costs for women and men, respectively). The prevalence rates for overweight and obesity in Brazil differed little between sexes, and women demonstrated almost half of PAR for selected diseases than men, suggesting a greater use of health care system among them.

The largest proportion of costs was attributable to the treatment of cardiovascular diseases (67%), most likely because of higher prevalence of coronary artery disease compared to the selected neoplasms. In a previous study carried out in Brazil, Sichieri et al. [14] also demonstrated that more than half of hospitalization costs were due to myocardial infarction and other ischemic heart diseases in public health system in Brazil.

Cardiovascular diseases and diabetes mellitus, both very common diseases with high morbidity and mortality rates, are responsible for a significant number of hospitalizations and high costs in Brazil [15,16]. Both conditions are related to obesity and probably its prevalence and severity could be reduced with the reduced obesity rates. Wang et al. studied the economic burden of the projected obesity trends in the United States of America (USA), and demonstrated that a hypothetical program that enables a 1% BMI reduction across the US population would avoid up to 2.1-2.4 million incident cases of diabetes, 1.4-1.7million cardiovascular diseases, and 73,000-127,000 cases of cancer [17].

The second group of diseases with the highest costs to the public health system was neoplasms. With the trend on increase prevalence of obesity along with the aging population, the increase in cancer cases and costs involved will be enormous.

Hospitalizations expenditures are often more important and contributed to the majority of costs to health systems, as presented in this analysis (approx 68% of total costs). The total expenditures related to all hospital admissions in Brazilian adult population (year 2010) amounted US$ 4.5 billion [18]. The estimate of obesity-related diseases expenditures accounted for 32.9% of these costs, and approximately 11% of these costs can be attributable to overweight and obesity. Likewise, Schieri et al. also studied the impact of obesity on hospitalizations in Brazil, limiting the analysis to fewer diseases but including workdays lost due to hospitalization. In their study overweight/obesity related costs accounted for at least 3-5% of total hospitalization costs in 2001 [14].

The estimated costs with obesity-related diseases are equivalent to 0.09% of the Brazilian gross domestic product in 2010 [19]. Similarly a recent review for Europe which encompassed both direct and indirect costs estimated obesity-related costs to range from 0.09% to 0.61% of total annual gross domestic income in Western European countries [20]. In United Kingdom (UK), a review of costs studies of overweight and obesity demonstrated that both conditions were responsible for 7.3% of morbidity and mortality in the UK, contributing over £3 billion to the direct health cost burden to the public health system (4.6% of total expenditure in 2002) [21]. In Korea total costs represented about 0.22% of the gross domestic product and 3.7% of the national health care expenditures in 2005 [22]. During the past 20 years, there has been a dramatic increase in obesity in the United States totalled and in 2010, no state had a prevalence of obesity less than 20% and 12 of them had a prevalence of 30% or more [23]. The medical care costs of obesity in the United States totalled about US$147 billion in 2008 [24]. A recent review provides an overview research on the economic impact of the obesity epidemic with a broader view of the issue. Besides the high direct medical costs (obese spent 36-100% more than normal-weight controls), absenteeism and presenteeism were 1,5 times higher, and an increase in disability payments and disability insurance premiums were shown [25].

There is widespread agreement across this literature that the medical costs associated with obesity are substantial; however, there are important differences between the studies. Possible factors affecting these differences in estimates of costs are: methodology, categories of costs included (direct medical costs for diagnosis and treatment, indirect costs, non medical costs), definitions of weight categories, age groups, and data sources. These discrepancies make it difficult to compare results in different settings.

Some limitations on methods used in this study are worthy of further consideration. Firstly we restricted our analysis to meta-analysis of prospective studies and case–control studies carried out in countries other than Brazil, since no data are available on relative risks based on Brazilian cohorts. So, attributable risks may not reflect the real burden of the diseases in the country. Thus, our assumption is that the relative risks found in international studies could be applied to the Brazilian population. Secondly, in order to interpret PAR as the proportion of cases that could be prevented if the exposure were eliminated, we have to consider that confounding factors were controlled when relative risks were estimated. Thirdly, the overweight and obesity prevalence rates obtained from a Brazilian survey were based on self-reported weight and height, information that can be easily biased. Lastly, DATASUS is an administrative database designed with the purpose of operating the payment for hospitalizations, not for epidemiological purposes. Many limitations of this data can be raised as the quality of input data, coverage (60 to 70%), fraud, duplication of data, among others.

## Conclusion

This study provides a comprehensive estimate of the economic impact of overweight and obesity-related diseases for the Brazilian public health system. The knowledge of these costs will be useful for future economic analysis of preventive and treatment interventions, such as education programs or new drugs. This may help reduce obesity-attributable growth of health care expenditures in Brazil.

## Abbreviations

BMI, Body mass index; SUS, Brazilian health system; PAR, Population attributable risk; RR, Relative risk; DATASUS, National health database; UK, United Kingdom; USA, United States of America; WHO, World Health Organization.

## Competing interests

The authors declare that they have no competing interests.

## Authors’ contributions

All authors read and approve the final manuscript. LB and DVA made contributions to conception, design, analysis, and interpretation of data; were involved in drafting the manuscript and gave final approval of the version to be published. ESFC, LAB, GAA, TAM and CRP made contributions to acquisition, analysis, and interpretation of data; were involved in drafting the manuscript and gave final approval of the version to be published.

## Pre-publication history

The pre-publication history for this paper can be accessed here:

http://www.biomedcentral.com/1471-2458/12/440/prepub
